# Comparison of Riboflavin and Toluidine Blue O as Photosensitizers for Photoactivated Disinfection on Endodontic and Periodontal Pathogens *In Vitro*

**DOI:** 10.1371/journal.pone.0140720

**Published:** 2015-10-15

**Authors:** Henrik Krarup Nielsen, Javier Garcia, Michael Væth, Sebastian Schlafer

**Affiliations:** 1 Department of Dentistry, HEALTH, Aarhus University, Aarhus, 8000, Denmark; 2 Department of Biostatistics, HEALTH, Aarhus University, Aarhus, 8000, Denmark; Massachusetts General Hospital, UNITED STATES

## Abstract

Photoactivated disinfection has a strong local antimicrobial effect. In the field of dentistry it is an emerging adjunct to mechanical debridement during endodontic and periodontal treatment. In the present study, we investigate the effect of photoactivated disinfection using riboflavin as a photosensitizer and blue LED light for activation, and compare it to photoactivated disinfection with the widely used combination of toluidine blue O and red light. Riboflavin is highly biocompatible and can be activated with LED lamps at hand in the dental office. To date, no reports are available on the antimicrobial effect of photoactivated disinfection using riboflavin/blue light on oral microorganisms. Planktonic cultures of eight organisms frequently isolated from periodontal and/or endodontic lesions (*Aggregatibacter actinomycetemcomitans*, *Candida albicans*, *Enterococcus faecalis*, *Escherischia coli*, *Lactobacillus paracasei*, *Porphyromonas gingivalis*, *Prevotella intermedia* and *Propionibacterium acnes*) were subjected to photoactivated disinfection with riboflavin/blue light and toluidine blue O/red light, and survival rates were determined by CFU counts. Within the limited irradiation time of one minute, photoactivated disinfection with riboflavin/blue light only resulted in minor reductions in CFU counts, whereas full kills were achieved for all organisms when using toluidine blue O/red light. The black pigmented anaerobes *P*. *gingivalis* and *P*. *intermedia* were eradicated completely by riboflavin/blue light, but also by blue light treatment alone, suggesting that endogenous chromophores acted as photosensitizers in these bacteria. On the basis of our results, riboflavin cannot be recommended as a photosensitizer used for photoactivated disinfection of periodontal or endodontic infections.

## Introduction

Photoactivated disinfection (PAD) has proven to have a strong antimicrobial effect on a range of different microorganisms including bacteria and fungi [1–4]. PAD involves the activation of a photosensitizer (PS) by light of an appropriate wavelength, which generates reactive oxygen species (ROS) when molecular oxygen is present. During PAD, photosensitizing molecules attach to microbial cell structures and become excited to a high energy triplet state when light of an appropriate wavelength is captured by the chromophores of the PS. The triplet state PS may then react further via one or both of two pathways that lead to microbial killing: In type I reactions, triplet state PS reacts directly with microbial constituents by electron transfer. This produces radical ions which react with oxygen to generate ROS that can be detrimental to microbial membrane integrity [5, 6]. In type II reactions, triplet state PS transforms ground state molecular oxygen from the regular triplet configuration into the highly reactive singlet state. Singlet oxygen then causes oxidation of microbial constituents such as lipids, proteins and nucleic acids.

PAD is currently employed in several medical disciplines, including dentistry where it is emerging as an adjunct to mechanical procedures of biofilm debridement in the fields of endodontics and periodontology. The success of endodontic treatment is dependent on the complete removal of microorganisms from the intricate root canal system [7, 8], a condition that is not always achieved by conventional chemo-mechanical debridement [9–13]. Likewise, the outcome of periodontal therapy is dependent on the removal of pathogenic biofilms from the subgingival crevice.

For both diseases, PAD represents a promising therapeutic approach. Due to the high affinity of certain PS to bacterial membranes [14], the short half-life and the short diffusion paths of ROS [15], a strong and local disinfection can be achieved. Moreover, the unspecific mechanism of action renders the development of bacterial resistance unlikely [1, 14, 16].

Various light sources and PS are available for PAD. Most studies conducted in the field of dentistry use lasers to activate the PS [17–20], but excitation can as well be performed efficiently with less expensive conventional LED lamps [21–23]. Among the different PS employed in the field of dentistry, PAD with toluidine blue O (TBO) has been studied extensively, and it’s antimicrobial effect on oral pathogens is well documented [21–31].

Riboflavin (RFV, vitamin B_2_) is a promising alternative photosensitizing molecule. It is a micronutrient and an intrinsic PS which generates ROS when irradiated with blue light [32]. It is highly biocompatible and can be activated with LED lamps at hand, used for curing composite, in the dental office. Owing to its advantageous and well known toxicological and pharmacokinetic properties [33–35], RFV is labeled ‘generally recognized as safe’ by the FDA [36].

At present, literature on the effect of PAD using RFV/blue light to kill bacteria is sparse [37], and no reports focus on oral microorganisms. We therefore investigate the effect of PAD using RFV/blue light on a range of different endodontic and periodontal pathogens in planktonic suspension, and compare it to the effect of PAD using a combination of TBO and red light.

## Materials and Methods

### Microorganisms and culture conditions

Organisms included in the study were *Aggregatibacter actinomycetemcomitans* (HK915), *Candida albicans* (NCO 09001; = ATCC 11775), *Enterococcus faecalis* (DSM 20478), *Escherichia coli* (ATCC 11775), *Lactobacillus paracasei* (DSM 5622), *Porphyromonas gingivalis* (ATCC 33277), *Prevotella intermedia* (CCUG 24041) and *Propionibacterium acnes* (DSM 1897).

*A*. *actinomycetemcomitans* was cultivated on chocolate agar (Statens Serum Institut, Copenhagen, Denmark) in air enriched with 5% CO2; *C*. *albicans*, *E*. *faecalis*, *E*. *coli* and *L*. *paracasei* were grown on blood agar (Statens Serum Institut, Copenhagen, Denmark) under aerobic conditions; *P*. *acnes* was grown on blood agar under anaerobic conditions; *P*. *gingivalis* and *P*. *intermedia* were cultivated anaerobically on modified Columbia blood agar (Statens Serum Institut, Copenhagen, Denmark). All microorganisms were grown at 36.5°C.

Prior to experimental use, all organisms were grown in liquid culture until late exponential phase. *A*. *actinomycetemcomitans*, *P*. *gingivalis*, *P*. *intermedia* and *P*. *acnes* were cultivated in plaque medium [38]; *C*. *albicans*, *E*. *faecalis*, *E*. *coli* and *L*. *paracasei* were grown in tryptic soy broth (Scharlab, Barcelona, Spain). See S1 Table for details.

### Light sources and photosensitizers

Two LED lamps were used: FotoSan 630 LAD pen, emitting light in the red spectrum with a power peak at 630 nm, and FlashMax P3 460 emitting light in the blue spectrum with a power peak at 460 nm. Relative spectral power distributions are shown in S1 Fig. Both lamps were equipped with short, cone-shaped conductive tips (planktonic tip, Ø 4 mm at the cone end) during treatment of microorganisms.

Photosensitizers used were watery solutions of TBO, used in conjunction with FotoSan 630 LAD pen, and RFV, used in conjunction with FlashMax P3 460. Both photosensitizers were prepared to a concentration of 266 μmol L^-1^ and stored in the dark until experimental use. Light sources and photosensitizers were provided by CMS Dental (Copenhagen, Denmark).

### PAD treatment

Microorganisms were centrifuged for 5 min at 5.000 rpm and washed once in 0.9% sterile saline. For each organism, optical density measurements (Novaspec II, Pharmacia Biotech, Cambridge, England; 550 nm) were calibrated to cell counts in a microscopic counting chamber (Bürker-Türk, Glaswarenfabrik Karl Hecht “Assistent”, Sondheim/Rhön, Germany), and cell concentrations were adjusted to 10^7^-10^8^ mL^-1^ spectrophotometrically (see S1 Table).

For both photosensitizers (P) and the respective red or blue light sources (L), four different treatments were carried out on each organism. 1) PAD treatment (P+L+): Bacterial/fungal suspensions were vortexed for 30 s, after which 60 μL of bacterial suspension and an equal volume of photosensitizer solution (final concentration: 133 μmol L^-1^) were transferred to the lid (area: 0.64 cm^2^) of a sterile 1.5 mL Eppendorf tube and mixed with a sterile pipette. One minute after application of the photosensitizer, the suspension was irradiated for 1 min with the corresponding light source. During the irradiation of planktonic cells, the light tip was mounted and the cone end was held just above the suspension at the level of the lid entrance; 1 mm from the surface of the bacterial suspension. For both light sources, an energy dose of 24 J was delivered to the suspension (power: 0.4 W; fluence: 37.7 J/cm^2^; fluence rate: 0.63 W/cm^2^). 2) Negative control treatment (P-L-): Microbial suspensions were mixed with 60 μL of 0.9% sterile saline and the light source was placed in the irradiation position for 1 min without emitting light. 3) Photosensitizer alone (P+L-): Microbial suspensions and photosensitizer solution were mixed and the corresponding light source was held in irradiation position for 1 min without irradiating. 4) To test the effect of the light sources alone (P-L+), microbial suspensions and sterile saline were mixed and irradiated for 1 min. The same microbial parent suspension was used for each series of four treatments.

Immediately after treatment, samples of 100 μL were removed from the lids, subjected to serial dilution and cultivated on agar plates. Counts of colony-forming units (CFU) were determined on at least three replicate agar plates. All experiments were performed in duplicate.

To determine the effect of irradiation time on bacterial killing, suspensions of *E*. *faecalis* were subjected to PAD using different irradiation intervals. 60 μL of bacterial suspension and 60 μL of either TBO or RFV were mixed in Eppendorf lids and irradiated for 0 s, 10 s, 30 s, 1 min or 2 min with the corresponding light source. Again, the same microbial parent suspension was used. Serial dilution, plating and counting of CFU were performed as previously described. The experiments were performed in duplicate.

### Statistical analysis

The CFU count data were assumed to follow a Poisson distribution with a mean inversely proportional to the dilution factor. Data from each organism were analyzed separately by Poisson regression methodology using robust standard errors to account for a possible overdispersion. For both PAD systems (RFV/blue light, TBO/red light) the effect of each treatment (P+L+, P+L-, P-L+) was expressed as a survival fraction with a 95% confidence interval relative to the negative control (P-L-). To compare PAD with RFV/blue light (PAD_RFV_) to PAD with TBO/red light (PAD_TBO_), an interaction term was included in the regression model. The interaction term represented the ratio of the survival fractions for PAD_TBO_ relative to the survival fraction for PAD_RFV_. In the analysis of the time series data the treatment effect (P+L+ relative to P-L-) was also estimated by Poisson regression as a survival fraction for each PAD system and each irradiation period. All survival fractions were converted to log_10_ reductions. For all calculations, p-values below 0.05 were considered as statistically significant and p-values below 0.001 as statistically highly significant.

## Results

Overall, PAD using RFV/blue light (PAD_RFV_) killed less microorganisms than PAD with TBO/red light (PAD_TBO_). In planktonic suspensions of *C*. *albicans*, *E*. *coli* and *E*. *faecalis*, PAD_RFV_ did not reduce the amount of viable cells significantly. Moderate effects of PAD_RFV_ were observed for *A*. *actinomycetemcomitans* and *L*. *paracasei*, with mean log_10_ reductions of 1.11 and 1.36, respectively (p<0.001). A minor effect of PAD_RFV_ was observed for *P*. *acnes* (0.29 log_10_ reduction, p<0.001), but treatment with blue light alone led to a similar reduction (0.21 log_10_ reduction, p<0.001).

PAD_RFV_ only achieved full kills in the cases of *P*. *gingivalis* and *P*. *intermedia* (p<0.001). However, both species were also eradicated by 1 min of irradiation with blue light alone (p<0.001).

Experiments with different irradiation times showed that the effect of PAD_RFV_ on *E*. *faecalis* increased slightly with longer irradiation times. 0.22 log_10_ reduction was achieved after 10 s, 0.25 log_10_ reduction after 30 s, 0.37 log_10_ reduction after 1 min and 0.40 log_10_ reduction after 2 min.

When PAD_TBO_ was performed, total microbial killing was observed of all the species included. The effect of PAD_TBO_ was significantly stronger than the one of PAD_RFV_ for all organisms (p<0.001), except for *P*. *intermedia* and *P*. *gingivalis*, where PAD_RFV_ resulted in complete eradication, too.

Experiments with different irradiation times showed that *E*. *faecalis* was eradicated completely after 30 s of irradiation, and even 10 s of irradiation led to a 5.27 log_10_ reduction (p<0.001).

**Fig 1:**
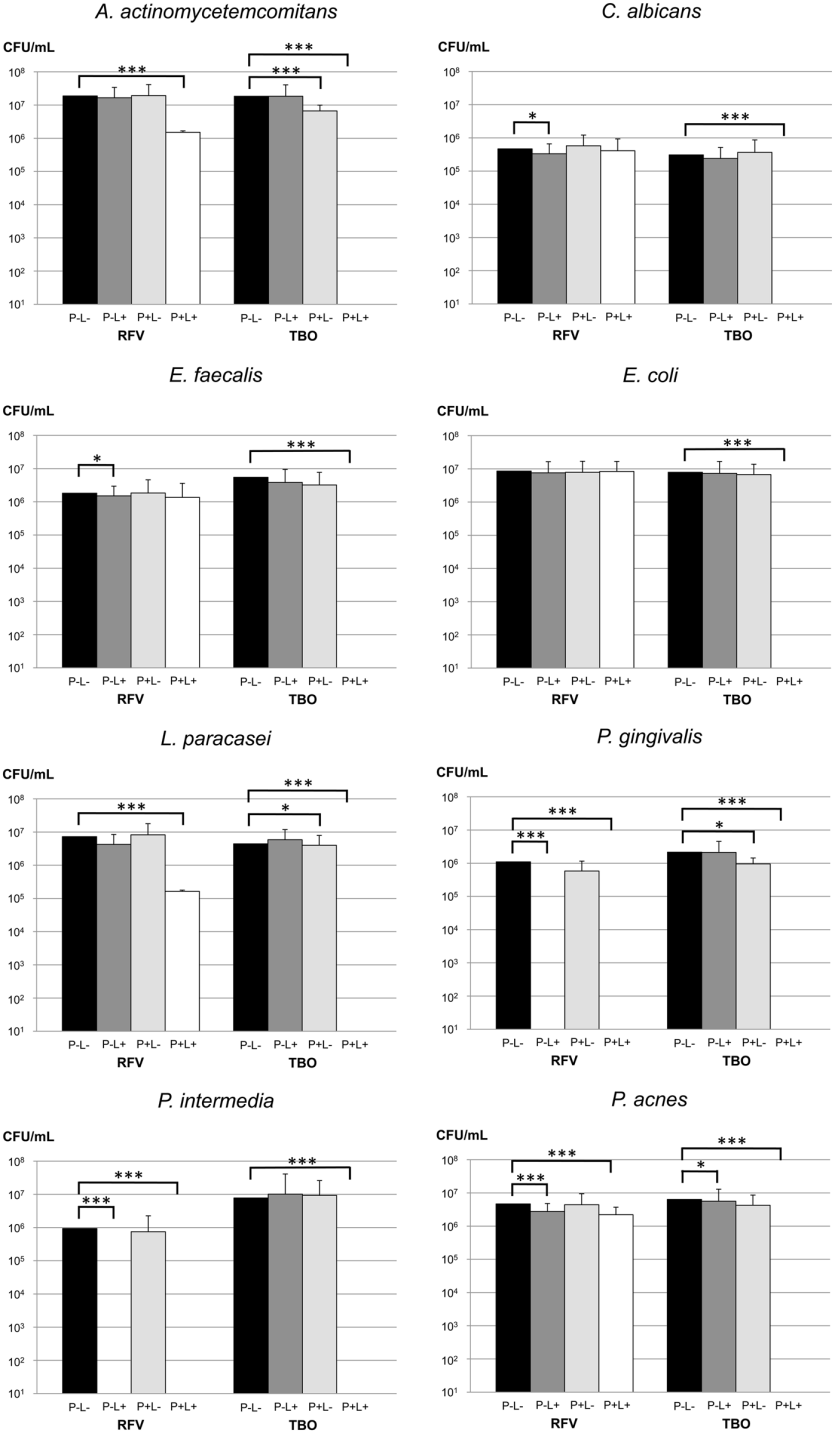
Effect of PAD using riboflavin (RFV)/blue light or toluidine blue O (TBO)/red light on selected microorganisms. For both PAD systems, CFU/mL of all species were determined after PAD treatment (P+L+), treatment with light alone (P-L+) and treatment with the photosensitizer alone (P+L-) and compared to negative control treatment (P-L-). * p<0.05; *** p<0.001.

TBO alone had a moderate yet significant effect on *A*. *actinomycetemcomitans* (0.46 log_10_ reduction, p<0.001) and P. gingivalis (0.69 log_10_ reduction, p = 0.001). Moreover, slight but statistically significant reductions were observed for treatment with TBO alone on *L*. *paracasei* (0.06 log_10_ reduction, p = 0.01) and treatment with blue light alone on *C*. *albicans* (0.05 log_10_ reduction, p = 0.04) and *E*. *faecalis* (0.09 log_10_ reduction, p = 0.02). All results are presented in Fig 1, S2 Table and S2 Fig.

## Discussion

On a theoretical plane, Riboflavin offers some advantages as a photosensitizer for photoactivated disinfection. It is a highly biocompatible molecule that can be used intraorally without reservation [36]. With its faint yellow color, it does not stain the hard tissues of the tooth as severely as TBO, which is advantageous in aesthetically important regions. Moreover, it can be excited with blue light emitting LED lamps at hand in the dental office. Implementing PAD with Riboflavin and blue light in dental treatments would thus require minimal effort.

It has previously been shown that RFV can be excited with blue light emitting LED lamps used for composite curing and that ROS are generated [32]. However, our study clearly shows that the antimicrobial effect of PAD with RFV and blue light cannot compete with the one of PAD using TBO and red light.

PAD_RFV_ left *C*. *albicans*, *E*. *coli* and *E*. *faecalis* unaffected, and it only had a moderate effect of less than 2 log reductions on *A*. *actinomycetemcomitans*, *L*. *paracasei* and *P*. *acnes* when compared to negative control treatment. The irradiation time series experiments performed on *E*. *faecalis* showed a tendency to a more pronounced antibacterial effect when cultures were irradiated for 2 min. Even longer irradiation times are likely to increase the antimicrobial effect of PAD with RFV and blue light, but they would be inacceptable in a dental clinical setting and were therefore not tested. PAD_RFV_ with longer exposure times might still be tested in other medical disciplines where the sites of infection are more easily accessible and treatment does not require the continuous presence of health care personnel [33, 37, 39–41].

Interestingly, PAD_RFV_ had a strong antibacterial effect on *P*. *gingivalis* and *P*. *intermedia*. Within one minute of irradiation, full kills were achieved for both organisms. However, control treatment with irradiation alone equally resulted in full kills of both species. *P*. *gingivalis* and *P*. *intermedia* belong to the black pigmented *Bacteroides* group that possesses the endogenous chromophores μ-oxo bisheme and hematin [42, 43]. Their respective absorption maxima being situated at 393 nm [44] and 398 nm [45], it is likely that μ-oxo bisheme and hematin acted as photosensitizers and caused bacterial killing in the experiments [46–48]. The same mechanism likely explains the moderate effect of blue light on *P*. *acnes*, which equally produces intracellular photosensitizing porphyrins, mainly coproporphyrin III [49]. All three organisms have previously been shown to be susceptible to blue light treatment. Effects observed on *P*. *acnes* were moderate [50, 51], while stronger effects, comparable to the ones we observed, were reported for treatment of *P*. *gingivalis* and *P*. *intermedia* with light doses comparable to the ones used in the present study [46, 52, 53].

By comparison, PAD_TBO_ had a strong effect on all eight investigated organisms. Full kills were achieved within the rather short irradiation time of one minute, and the time series experiments conducted with planktonic cultures of *E*. *faecalis* show that even shorter irradiation times of 30 s (full kill) or 10 s (3.7 log_10_ reduction) are adequate to obtain a powerful antibacterial effect. It is important to point out that an LED light source and not a laser was used for treatment. Most studies investigating the antibacterial effect of PAD_TBO_ employ lasers that cost more than LED lamps and are subjected to strong power output limitations to avoid causing damage to adjacent tissues. Comparison of treatment results from the present study with results from other studies obtained with laser light shows that LED light sources should be considered as cheaper and safer alternatives for PAD [29, 54–56].

Unlike blue light, treatment with red light alone did not result in strong reductions for any of the tested organisms. Previous studies have reported moderate effects of red light on *P*. *acnes*, *P*. *intermedia* and *P*. *gingivalis* [57, 58], but absorption by endogenous porphyrins in the red spectrum is comparatively low [43], and compared to blue light, higher energy doses are required to achieve antimicrobial effects [46].

The poor effect of PAD_RFV_ may be explained by a lower ROS production from RFV compared to TBO [32]. Moreover, the uncharged state of RFV may be unfavorable. It has been reported that cationic PS such as TBO show a better interaction with the Gram-negative cell membrane, which allow for a stronger antimicrobial effect [14]. The present study, however, did not demonstrate an inferior effect of PAD_RFV_ on Gram-negatives compared to Gram-positives.

While the effect of PAD_TBO_ on pathogens in planktonic suspension is strong, a number of problems encountered in clinical situations may compromise the effect *in vivo*: 1. Biofilms, and especially multi-species biofilms, are more resistant to treatment than planktonic cells [59]. 2. The propagation of light to the site of infection is hampered by local factors (deep periodontal pockets, intricate root canal anatomy). 3. The photosensitizer might be inactivated and ROS absorbed by molecules in biological fluids (blood, pus, gingival crevicular fluid, saliva). 4. Low availability of oxygen in deep periodontal pockets and in root canals might limit the production of ROS and thus the effect of PAD.

## Conclusion

The present study shows that Riboflavin is not suitable as a photosensitizer for use in endodontic or periodontal therapy. Limited microbial kills of PAD using riboflavin/blue light were observed for most of the investigated species within the short irradiation times that are practicable in a dental clinical setting. Photoactivated disinfection with toluidine blue O and red light provided by an LED lamp, on the other hand, had an excellent antimicrobial effect on all investigated species and merits further research. LED light sources should be considered as cheaper and safer alternatives to lasers for photoactivated disinfection.

## Supporting Information

S1 Fig Relative spectral power distribution of the LED lamps. Power peaks of the red (**A**) and blue (**B**) LED lamps match the excitation maxima of toluidine blue O (630 nm) and riboflavin (460 nm).
(TIF)

S2 Fig Effect of PAD on *E*. *faecalis* using different irradiation times. SF = survival fraction; PAD = photoactivated disinfection; RFV = riboflavin; TBO = toluidine blue O.
(TIF)

S1 Table Growth conditions of the employed organisms. Optical densities corresponding to cell concentrations of 10^7^-10^8^ mL^-1^.
(DOCX)

S2 Table Effect of PAD compared to negative control treatment. P+L+ = photosensitizer, light (treatment); P+L- = photosenzitizer, no light; P-L+ = sterile saline; Log_10_ = log_10_ reduction; SF = survival fraction; CI = confidence interval; RFV = riboflavin; TBO = toluidine blue O; * = statistically significant reduction.
(DOCX)
