# Supplementary figures and images for: Comparison of Riboflavin and Toluidine Blue O as Photosensitizers for Photoactivated Disinfection on Endodontic and Periodontal Pathogens In Vitro
Source: PLoS One. 2015 Oct 15;10(10):e0140720. doi: 10.1371/journal.pone.0140720 (PMC4607437; doi:10.1371/journal.pone.0140720)

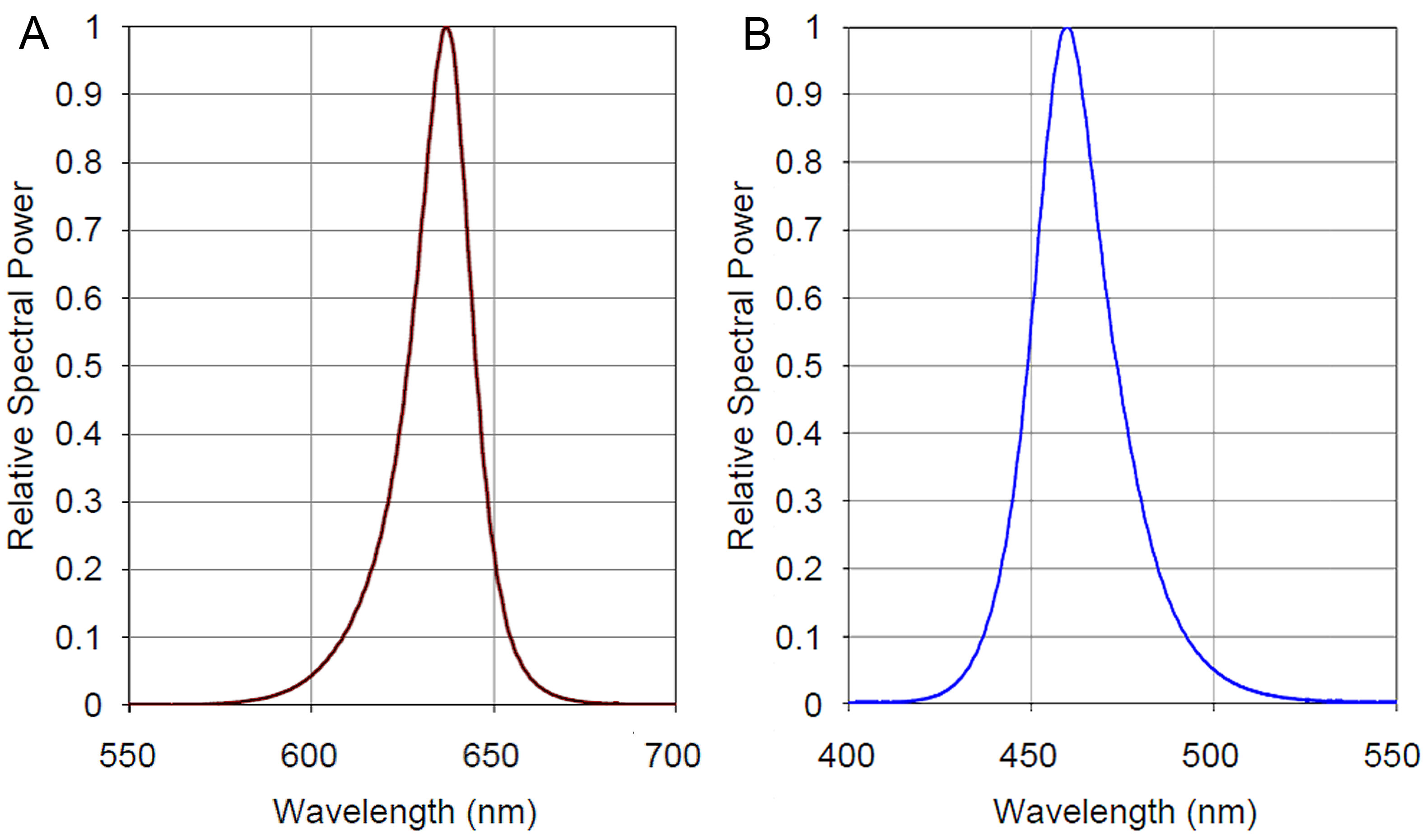

Supplement: S1 Fig — Power peaks of the red (A) and blue (B) LED lamps match the excitation maxima of toluidine blue O (630 nm) and riboflavin (460 nm). (TIF) [file pone.0140720.s001.tif]

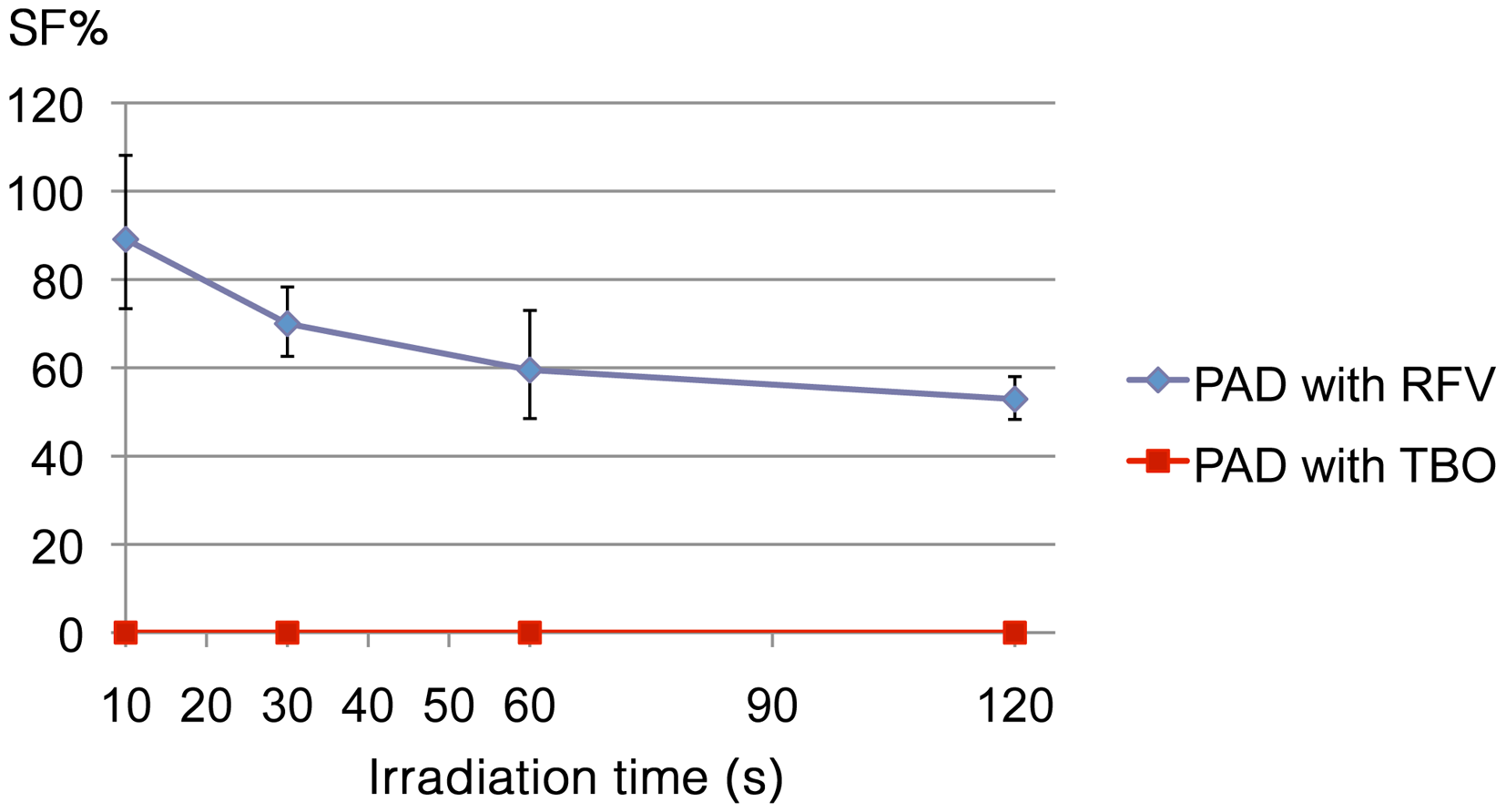

Supplement: S2 Fig — SF = survival fraction; PAD = photoactivated disinfection; RFV = riboflavin; TBO = toluidine blue O. (TIF) [file pone.0140720.s002.tif]
