# Supplementary material for: Comparison of Riboflavin and Toluidine Blue O as Photosensitizers for Photoactivated Disinfection on Endodontic and Periodontal Pathogens In Vitro
Source: PLoS One. 2015 Oct 15;10(10):e0140720. doi: 10.1371/journal.pone.0140720 (PMC4607437; doi:10.1371/journal.pone.0140720)
Supplement: S1 Table — Optical densities corresponding to cell concentrations of 107-108 mL-1. (DOCX) [file pone.0140720.s003.docx]

| **S1 Table.** Growth conditions of the employed organisms | | | |
| --- | --- | --- | --- |
| **Species** | **Temp (°C)** | **Time (h)** | **OD_550_** |
| *A. actinomycetemcomitans* | 37.0 | 24.0 | 0.35 |
| *C. albicans* | 20.0 | 21.0 | 0.80 |
| *E. faecalis* | 15.0 | 24.0 | 0.20 |
| *E. coli* | 28.0 | 20.0 | 0.40 |
| *L. paracasei* | 28.0 | 23.0 | 0.30 |
| *P. gingivalis* | 35.0 | 48.0 | 0.20 |
| *P. intermedia* | 37.0 | 20.0 | 0.65 |
| *P. acnes* | 35.0 | 23.0 | 0.20 |
| Optical densities corresponding to cell concentrations of 10^7^-10^8^ mL^-1^. | | | |
|  |  |  |  |
