# Supplementary material for: Comparison of Riboflavin and Toluidine Blue O as Photosensitizers for Photoactivated Disinfection on Endodontic and Periodontal Pathogens In Vitro
Source: PLoS One. 2015 Oct 15;10(10):e0140720. doi: 10.1371/journal.pone.0140720 (PMC4607437; doi:10.1371/journal.pone.0140720)
Supplement: S2 Table — P+L+ = photosensitizer, light (treatment); P+L- = photosenzitizer, no light; P-L+ = sterile saline; Log10 = log10 reduction; SF = survival fraction; CI = confidence interval; RFV = riboflavin; TBO = toluidine blue O; * = statistically significant reduction. (DOCX) [file pone.0140720.s004.docx]

| **S2 Table.** Effect of PAD compared to negative control treatment | | | | | | | | | | | | | |
| --- | --- | --- | --- | --- | --- | --- | --- | --- | --- | --- | --- | --- | --- |
|  |  | **P+L+** | | | | **P+L-** | | | | **P-L+** | | | |
|  | **Species** | **Log_10_** | **SF%** | **95% CI** | **p-value** | **Log_10_** | **SF%** | **95% CI** | **p-value** | **Log_10_** | **SF%** | **95% CI** | **p-value** |
| RFV/ blue light | *A. actinomycetem.* | 1.11* | 7.8 | (6.0;10.2) | <0.001 | - | 104.2 | (94.7;114.7) | 0.40 | 0.07 | 84.5 | (67.9;105.2) | 0.13 |
|  | *C. albicans* | - | 114.8 | (103.6;127.1) | 0.01 | - | 103.6 | (96.6;111.0) | 0.33 | 0.05* | 88.2 | (78.4;99.2) | 0.04 |
|  | *E. faecalis* | 0.01 | 98.8 | (59.5;163.9) | 0.96 | - | 103.2 | (71.4;149.3) | 0.87 | 0.09* | 81.6 | (69.1;96.4) | 0.02 |
|  | *E. coli* | 0.03 | 94.3 | (88.3;100.7) | 0.08 | - | 103.2 | (92.6;115.0) | 0.57 | - | 104.4 | (93.9;116.1) | 0.42 |
|  | *L. paracasei* | 1.36* | 4.4 | (2.4;8.3) | <0.001 | 0.03 | 94.3 | (76.1;116.9) | 0.60 | 0.08 | 83.2 | (69.4;99.7) | 0.05 |
|  | *P. gingivalis* | ∞* | 0 | - | <0.001 | 0.17 | 68.3 | (46.9;99.3) | 0.05 | 4.70* | 0.002 | (0.000;0.008) | <0.001 |
|  | *P. intermedia* | 4.70* | 0.002 | (0.000;0.005) | <0.001 | 0.03 | 93.8 | (43.8;201.1) | 0.87 | ∞* | 0 | - | <0.001 |
|  | *P. acnes* | 0.29* | 50.8 | (37.7;68.5) | <0.001 | 0.02 | 95.5 | (80.4;113.5) | 0.60 | 0.21* | 61.4 | (51.0;73.8) | <0.001 |
|  |  |  |  |  |  |  |  |  |  |  |  |  |  |
| TBO/ red light | *A. actinomycet.* | ∞* | 0 | - | <0.001 | 0.46* | 34.3 | (23.7;49.5) | <0.001 | - | 104.3 | (90.0;120.9) | 0.58 |
|  | *C. albicans* | ∞* | 0 | - | <0.001 | - | 123.4 | (111.0;137.3) | <0.001 | 0.05 | 88.9 | (69.1;114.3) | 0.36 |
|  | *E. faecalis* | ∞* | 0 | - | <0.001 | 0.02 | 95.7 | (65.3;140.0) | 0.82 | - | 107.2 | (78.9;145.7) | 0.66 |
|  | *E. coli* | ∞* | 0 | - | <0.001 | 0.03 | 92.3 | (80.6;105.6) | 0.24 | - | 111.3 | (96.4;128.6) | 0.15 |
|  | *L. paracasei* | ∞* | 0 | - | <0.001 | 0.06* | 87.4 | (78.6;97.2) | 0.01 | - | 103 | (88.9;119.3) | 0.69 |
|  | *P. gingivalis* | ∞* | 0 | - | <0.001 | 0.69* | 20.6 | (8.3;51.0) | 0.001 | 0.02 | 95 | (78.3;115.2) | 0.60 |
|  | *P. intermedia* | ∞* | 0 | - | <0.001 | 0.54 | 28.6 | (4.6;179.5) | 0.18 | 0.01 | 97.6 | (31.2;305.8) | 0.97 |
|  | *P. acnes* | ∞* | 0 | - | <0.001 | 0.04 | 91.6 | (81.6;102.9) | 0.14 | - | 115 | (101.4;130.3) | 0.03 |
| P+L+ = photosensitizer, light (treatment); P+L- = photosenzitizer, no light; P-L+ = sterile saline; Log_10_ = log_10_ reduction; SF = survival fraction; CI = confidence interval; RFV = riboflavin; TBO = toluidine blue O; * = statistically significant reduction. | | | | | | | | | | | | | |
|  |  |  |  |  |  |  |  |  |  |  |  |  |  |
